# Population Genetic Structure of the Endangered Kaiser’s Mountain Newt, *Neurergus kaiseri* (Amphibia: Salamandridae)

**DOI:** 10.1371/journal.pone.0149596

**Published:** 2016-02-26

**Authors:** Hossein Farasat, Vahid Akmali, Mozafar Sharifi

**Affiliations:** Razi University Center for Environmental Studies, Department of Biology, Faculty of Science, Razi University, Bagheabrisham 6714967346, Kermanshah, Iran; Instituto de Higiene e Medicina Tropical, PORTUGAL

## Abstract

Species often exhibit different levels of genetic structuring correlated to their environment. However, understanding how environmental heterogeneity influences genetic variation is difficult because the effects of gene flow, drift and selection are confounded. We investigated the genetic variation and its ecological correlates in an endemic and critically endangered stream breeding mountain newt, *Neurergus kaiseri*, within its entire range in southwestern Iran. We identified two geographic regions based on phylogenetic relationships using Bayesian inference and maximum likelihood of 779 bp mtDNA (D-loop) in 111 individuals from ten of twelve known breeding populations. This analysis revealed a clear divergence between northern populations, located in more humid habitats at higher elevation, and southern populations, from drier habitats at lower elevations regions. From seven haplotypes found in these populations none was shared between the two regions. Analysis of molecular variance (AMOVA) of *N*. *kaiseri* indicates that 94.03% of sequence variation is distributed among newt populations and 5.97% within them. Moreover, a high degree of genetic subdivision, mainly attributable to the existence of significant variance among the two regions is shown (θ_CT_ = 0.94, P = 0.002). The positive and significant correlation between geographic and genetic distances (r = 0.61, P = 0.002) following controlling for environmental distance suggests an important influence of geographic divergence of the sites in shaping the genetic variation and may provide tools for a possible conservation based prioritization policy for the endangered species.

## Introduction

Distribution of genetic variation across animal and plant populations is under influence of the environmental heterogeneity which has long been recognized as an important factor in the evolution of fitness-related traits in the wild [1]. Such spatial structuring of intraspecific genetic diversity can also occur under both historical and current evolutionary processes such as geographic distances between populations [2], physical barriers to gene flow [3], and habitat fragmentation [4]. In many populations, gene flow tends to decrease with increasing geographic distances, resulting in an increase in genetic differentiation among individuals. Spatial genetic variation known as ‘isolation by distance’ [5] can be detected by analyzing the distribution of pair-wise estimates of genetic distances between individuals [6]. Such analysis can also demonstrate associations between genetic variation and environmental variation known as “isolation by environmental distance” [7]. Here, environmental characteristics (or ecological processes) may influence gene flow either by disrupting dispersal or by influencing survival (e.g. local adaptation) [8,9]. There are numerous studies that show spatial structures in genetic composition that are associated with “isolation by distance” (e.g.[10,11]), “isolation by environmental distance” (e.g.[12,13]) or “isolation by ecological processes” (e.g.[8,9,14]) for various taxa under different circumstances.

Population divergence generated by geographical or environmental isolation as a consequence of natural or anthropological variation are normally well documented in the form of demonstrating association between genetic variation and geographic or environmental distances [1,2]. However, exploring the casual role of environmental or geographical factors in producing genetic differentiation in an isolated population and assessing their importance relative to that of other factors is difficult [15] because the interactions among various factors cannot always be detected by isolation-by-distance association [16]. Explicit evaluation of environmental impact on the spatial distribution of genetic variation is now possible by employing good molecular markers, geographic information system (GIS) data and spatial statistics [17,18,19]. Using this approach it is possible to assess how various evolutionary processes such as gene flow and connectivity between populations, neutral and selective processes and the extent of local adaptation are affected by specific landscape and environmental features [20].

In recent years, combining genetic, geographic and ecological data have been used for various conservation purposes including delimiting or defining population distinctiveness of various types [21], mapping suitable habitat for endangered species [22], selecting re-introduction sites [23], restoring native populations in their natural habitat [24], and designing conservation and management plans [25]. However, ecological and spatial data concerning distribution and ecological roles of endangered species are often sparse. *N*. *kaiseri* is a poorly known amphibian that is restricted to the highlands of the Zagros Mountains in southwestern Iran. This mountain newt is endemic to the first order streams at elevations ranging between 800 and 1500 m a.s.l. [26]. *N*. *kaiseri* has been evaluated as a critically endangered species by International Union for Conservation of Nature (IUCN) criteria [27]. This species has also been amended to the Appendix I of the Convention to the International Trade to Endangered Species (CITES).

The main aims of this study were to (1) determine whether *N*. *kaiseri* populations exhibited genetic differentiation using traditional population genetics techniques (D-loop sequence analysis), (2) examine on the ecological differences found between the regions associated with populations that show genetic differentiations and (3) associate the pattern of genetic differences with spatial differences to provide tools for a possible conservation-based prioritization policy for the Kaiser’s mountain newt.

## Materials and Methods

### Ethics statement

The permit for collecting toe clips from live Kaiser’s mountain newts in 10 of 12 known breeding streams was issued by Kermanshah Department of Environment. None of the breeding streams are located in any of the four sanctuaries (National Park, Protected Regions, Natural Monument and Wildlife Refuge) under legal control of the Iranian Department of Environment. Meanwhile, these streams which are located at high elevation in remote parts of Zagros Mountains are not owned by private sectors therefore access to these areas doesn't require any permit. No approval was obtained for ethical conduct of the present study because laboratory and field studies in Iran are not directed by law.

### Study site and sampling

This study was conducted across the entire species’ natural distribution range in southern Zagros Mountains in Iran encompassing a minimum convex polygon of 789 km2 [26]. The localities are breeding streams generally dispersed with nearest neighbor distances among the known breeding localities average 11.84 km (range, 1.14–39.28 km). These streams are separated from one another by steep and rocky terrain at elevations between 930−1395 meters with vegetation cover of mature open oak woodland in the west and sparse scrubland or thin oak-pistachio woodland in the central area and east, thus potentially isolating many of the populations from each other [26].

**Fig 1:**
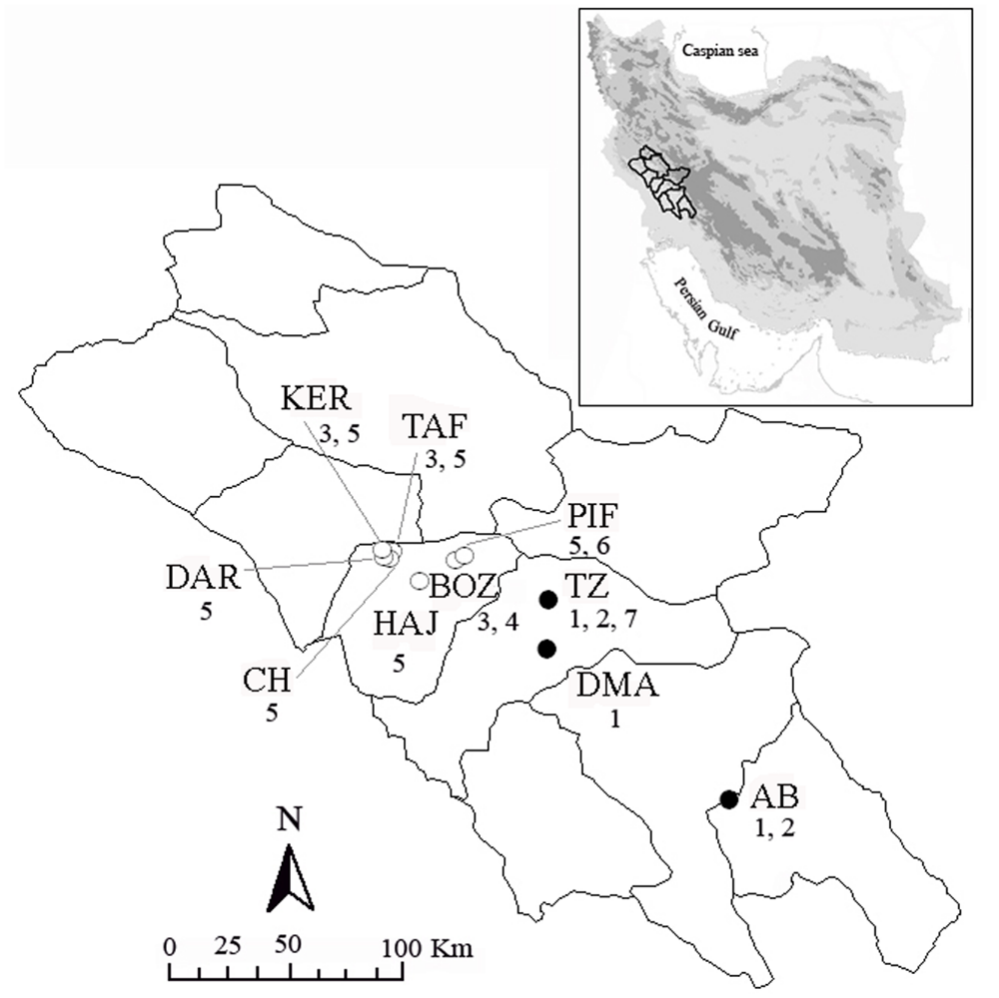
Map of sampling localities of *N*. *kaiseri* in the south-western Zagros Range, Iran for genetic analyses. Site IDs correspond to IDs in Table 1. Note that northern region populations are denoted with open circles and that southern region populations are denoted with closed circles. Numbers designate haplotypes found within each population. The polygons illustrate administrative boundaries.

We collected 114 samples (included: 111 *N*. *Kaiseri*) from 10 out of 12 known breeding streams throughout the range of *N*. *kaiseri* in Iran (Fig 1; Table 1). We captured adult newts with dip nets in their breeding stream. Knowing that tissue will regenerate, we removed a small section from the tail tip or clipping toe (i.e. second or third) and preserved in 70% ethanol. Scissor blades were disinfected with ethanol and flame between clippings. The newts were kept in small (30x30 cm) pools by putting up several stones at the sampling site for approximately two hours to see if toe amputation caused any visible side effect such as bleeding, and then released. We did not observe any apparent side effects following the amputations.

**Table 1:** Variable nucleotide positions and molecular diversity within the partial sequences (779 bp) of the D-loop region for 7 haplotypes of 111 *N*. *kaiseri* sampled from southern Zagros Range, Iran. New haplotypes (H1 to H7) are reported here.

Haplotype	Polymorphic site											Southern region			Northern region							Total
	3	10	93	367	445	447	506	511	515	518	599	AB^a^	TZ^b^	DMA^c^	DAR^d^	HAJ^e^	KER^f^	CH^g^	PIF^h^	BOZ^i^	TAF^j^	
1	**G**	**C**	**C**	**T**	**G**	**G**	**T**	**C**	**T**	**A**	**G**	11	4	12	-	-	-	-	-	-	-	27
2	**•**	**•**	**T**	**•**	**•**	**•**	**•**	**•**	**•**	**•**	**•**	1	2	-	-	-	-	-	-	-	-	3
3	**A**	**T**	**•**	**•**	**A**	**A**	**•**	**•**	**C**	**G**	**T**	-	-	-	-	1	1	-	-	11	4	17
4	**A**	**T**	**T**	**•**	**A**	**A**	**•**	**•**	**C**	**G**	**T**	-	-	-	-	-	-	-	-	1	-	1
5	**A**	**T**	**•**	**•**	**A**	**A**	**C**	**•**	**C**	**G**	**T**	-	-	-	11	10	10	11	8	-	8	58
6	**A**	**T**	**•**	**C**	**A**	**A**	**•**	**•**	**C**	**G**	**T**	-	-	-	-	-	-	-	1	-	-	1
7	**•**	**•**	**•**	**•**	**•**	**•**	**•**	**T**	**•**	**•**	**•**	-	4	-	-	-	-	-	-	-	-	4
Sample size												12	10	12	11	11	11	11	9	12	12	111
Haplotypes diversity (h)												0.17	0.71	0.00	0.00	0.18	0.18	0.00	0.22	0.17	0.48	0.65
Number of haplotype												2	3	1	1	2	2	1	2	2	2	7
Nucleotide diversity (π)												0.0002	0.0011	0.0000	0.0000	0.0002	0.0002	0.0000	0.0006	0.0002	0.0006	0.0047
Number of polymorphic sites												1										11
Number of transitions												1	2	-	-	1	1	-	2	1	1	10
Number of transversions												-	-	-	-	-	-	-	-	-	-	1
Number of parsimony informative sites												-	2	-	-	-	-	-	-	-	1	10
Average number of nucleotide difference												0.17	0.89	-	-	0.18	0.18	-	0.44	0.17	0.48	3.66

^a^: Abejdan;

^b^: Talezang;

^c^: Dej Mohamad Alikhan;

^d^: Daregol;

^e^: Hajibarikab;

^f^: Kerser;

^g^: Choobeh;

^h^: Pifeh;

^i^: Bozorgab;

^j^: Tafo.

Samples were collected on six occasions: late March 2011, early May 2011, late May 2011, mid-June 2011, late May 2012, and mid-June 2012. We determined the elevation and geographic coordinates of all sites using a Garmin GPS unit (GPSMAP 60CSx; Garmin International, New York, USA). We used ArcGIS 9.3 (ESRI, Redlands, California, USA) and Google Earth (Google, Inc., Mountain View, California, USA) to calculate the area of the convex polygon encompassing the localities and to determine linear distances between localities and the breeding streams where newts were observed.

### DNA isolation and sequencing

We extracted total genomic DNA from each sample using Tissue Kits (GenNetBio^™^), following the manufacturer’s instructions (Seoul, South Korea). For genetic analysis we used Polymerase Chain Reaction (PCR) to amplify a 779 base-pair fragment of the mitochondrial (mtDNA) D-loop region using primers L-Pro-ML 5′-GGCACCCAARGCCAAAATTCT-3′ and H-12S1-ML 5′- CAAGGCCAGGACCAAACCTTTA-3′ [28]. In the H-12S1-ML primer we used R nucleotide instead of G just for a single base. Polymerase chain reactions (PCRs) were carried out in a final volume of 25 μl containing optimized amounts of PCR water, 12.5 μl of Master Mix kit (Sinaclon, Iran), 0.5 μl of each primer (10μM), and 2–5 ng of genomic DNA template. PCR conditions were as follows: initial denaturation of 94°C for 4 min, 35 cycles of 95°C for 30 s, 49°C for 45 s and 72°C for 1 min; and a final extension 72°C for 7 min. Purification of PCR products and sequencing were commercially performed by Macrogen (Korea). Sequencing was performed with both primers mentioned above. The haplotype sequences obtained have been deposited to GenBank (Accession numbers KP748175- KP748182).

#### Genetic Data analysis

The resulting sequences of the D-loop fragment were edited manually and automatically aligned using the BioEdit v. 5.0.9 program [29]. The identity of the consensus sequences was assessed and confirmed using BLAST (http://www.ncbi.nlm.nih.gov/BLAST/) and 779 bp were included for the final alignment. The D-loop region sequences from *N*. *microspilotus*, *N*. *strauchii* and *Triturus karelinii* as closely related taxa were used as out-groups. *N*. *microspilotus* has recently been considered synonymous with *N*. *derjugini* [30]. Individual haplotypes were used to construct a Bayesian inference (BI) tree in MrBayes, version 3.1.2 [31] and Maximum likelihood (ML) with PHYML, version 3.0 [32]. The appropriate model for BI and ML analysis was selected with jModelTest, version 0.1.1 [33] using Akaike Information Criterion (AIC). The best fit model identified by AIC for phylogenetic reconstruction was TPM1uf+G.

In ML analysis a starting tree was obtained by BIONJ and nodal support was estimated from 1500 bootstrap replicates. Bayesian Inference was performed for 5,000,000 generations. After discarding 5000 sampled trees, a consensus tree with posterior probabilities was generated and visualized using the FigTree v1.3.1 [34]. In this method the same substitution model was used as the ML analyses. In both analyses, the tree was rooted with outgroups. To complement the tree-based approaches we also implemented 95% statistical parsimony haplotype network [35] using the program TCS implemented in NCPA version 1.1 [36].

Additionally, genetic diversity was estimated for all populations based on haplotype diversity (h) and nucleotide diversity (π). Values for the numbers of polymorphic sites, parsimony informative site, and the mean numbers of pairwise differences among sequences and number of transitions and transversions were also calculated using the software DnaSP version 4.0 [37] and Arlequin version 3.1 [38]. To investigate intraspecific divergence within *N*. *kaiseri* in different populations, genetic distances, net average distances and standard error estimates were computed using Kimura 2-parameter [39] with 1,000 bootstrap replicates using MEGA ver. 4 software package [40]. Uncorrected pairwise Kimura 2-parameter distances for 7 haplotypes that are recovered from our analyses were also calculated using the Kimura 2-parameter model.

To assess differences between northern and southern regions in nucleotide and haplotype diversities we used Independent-Sample *T*-Test (2- tailed). Molecular variance was assessed using separate analyses of molecular variance (AMOVA) with 10,000 permutations [41] at several possible population groupings of *N*. *kaiseri* in Arlequin version 3.1 [38]. First the analysis was performed considering only the population from northern region, then considering only the populations from southern region to assess the hierarchical genetic structure in each region. Second, the analysis was performed considering all populations and assigning them to the correspondent regions to assess the degree of differentiation among regions. Finally, the analysis was performed without considering regions, with all populations in one group. The different structures were assessed according to the degree of differentiation among regions (θ_CT_), among populations within regions (θ_SC_) and within populations (θ_ST_).

### Environmental data

Climate data for current conditions were obtained from the WorldClim database (downloaded from the WorldClim database; www.worldclim.org; [42], in the raster format at 30 arc-second resolution (0.93 × 0.93 = 0.86 km^2^ at the equator). Initially, the correlations among all 21 WorldClim bioclimatic variables and topographic variables for all locations were calculated to exclude the highly correlated ones (r>75), whilst keeping variables such as climatic averages and extremes. Second, we chose 12 of the bioclimatic variables to describe the ecological characteristics of the sampled stands. The 12 bioclimatic variables included averages, extremes and seasonal variation in precipitation and temperature, and the topographic variable altitude. The following 12 climatic variables were included in the final subset: annual mean temperature, isothermality, maximum temperature of warmest month, mean temperature of wettest quarter, mean temperature of driest quarter, mean temperature of warmest quarter, mean temperature of coldest quarter, precipitation of wettest month, precipitation seasonality, precipitation of wettest quarter, precipitation of coldest quarter and elevation (see Table 2).

**Table 2:** Hierarchical analysis of molecular variance (AMOVA) among mtDNA D-loop region sequences of *N*. *kaiseri* in different geographical groupings. Percentage of variation is provided for three hierarchical levels.

Structure	Source of variation	Variation (%)	Fixation indices	P-value
**North region**	Among populations/Within region	55.67	θST = 0.56	< 0.001
	Within populations	44.33	-	-
**South region**	Among populations/Within region	22.34	θST = 0.22	< 0.001
	Within populations	77.65	-	-
**Two regions (North and South)**	Among regions	94.31	θCT = 0.94	0.002
	Among populations/Within regions	2.69	θSC = 0.47	< 0.001
	Within populations	3.00	θST = 0.97	< 0.001
**The studied samples**	Among populations	94.03	θST = 0.94	< 0.001
	Within populations	5.97	-	-

### Ecological comparisons between regions

#### Principal Components Analysis (PCA)

We performed principal components analysis (PCA) to further investigate ecological differentiation within *N*. *kaiseri* distribution range. We used PCA to compare environmental data at occurrence points between regions using SPSS statistical package (version 15, SPSS Inc., Chicago, IL, USA). The multivariate procedures provide an intuitive interpretation. We extracted environmental data at each occurrence point of the 10 sampling populations and two remaining localities resulting in a total of seven occurrence points for the northern region and five points for the southern region, in ArcGIS. The relative contribution of each environmental parameter to the formation of clusters was then represented in a PCA distance biplot, and the magnitude and statistical significance of each variable among the occurrence clouds in the PCA graph were assessed. We evaluated environmental variable importance by correlating each variable with axis scores from the PCA ordination.

#### Correlation between genetic, geographic and environmental distances

We tested the isolation by distance among the studied samples by plotting the matrix of pairwise genetic differentiation index (θ_ST_) against geographic distance (in km) between pairs of populations [43]. To assess the statistical significance of the correlation between genetic and geographic distances across the entire range of the species, a Mantel test was performed. Euclidian distances between the populations were computed to obtain a matrix of environmental distances among the populations. Mantel tests were used to assess the extent to which the neutral genetic structure can be described by the environmental heterogeneity. We computed and tested the correlations between the matrix of pairwise genetic differentiation index (θ_ST_) and the matrix of environmental distances variables. In addition, a three-way Mantel test was applied between the matrix of pairwise genetic differentiation index (θ_ST_) and the matrix of environmental distances while accounting for geographical distances among populations. The significance level was assessed after 10,000 permutations as implemented in Arlequin version 3.1 [38].

## Results

### Genetic structure within and among populations and biogeographical regions

**Fig 2:**
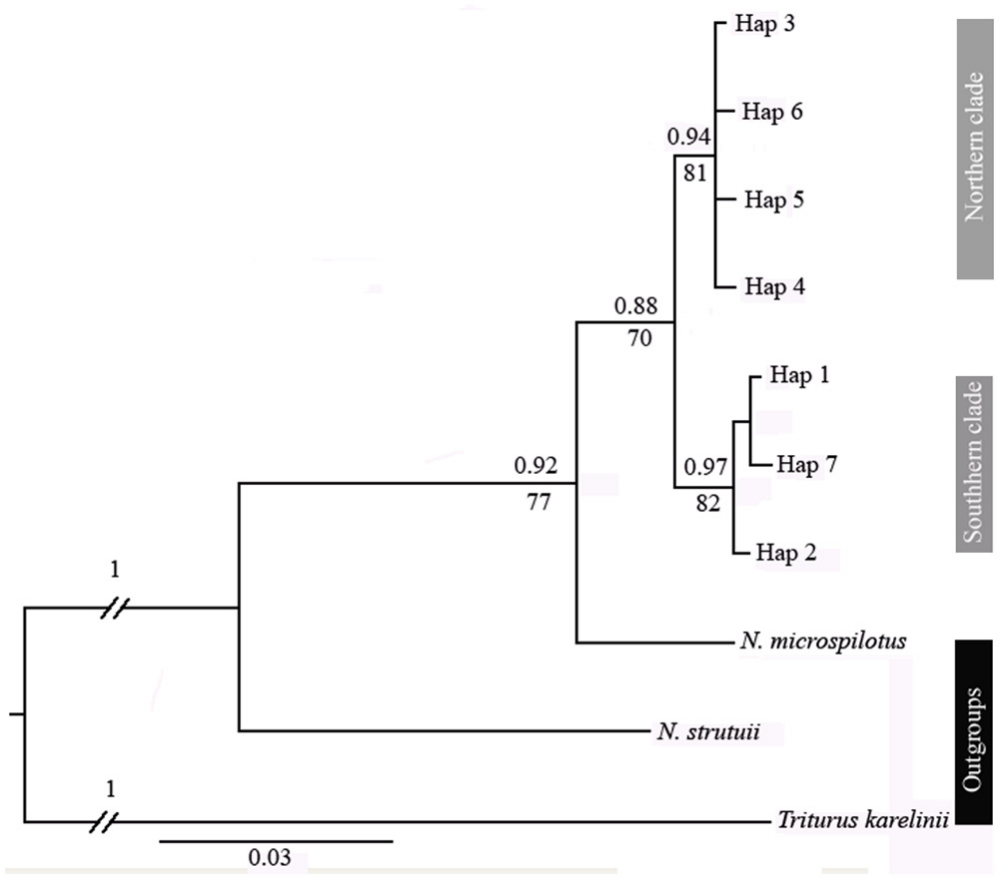
A majority-rule consensus tree of haplotypes of D-loop sequences for *N*. *kaiseri* samples plus outgroups, constructed by Bayesian inference (BI). Maximum likelihood topologies were identical. Numbers at the nodes refer to the bootstrap values in the Bayesian inference (above line) and Maximum likelihood (below line) analysis. The haplotype names refer to the sampling locations depicted in Fig 1.

We identified 7 unique haplotypes (Table 1 and Fig 2) from a total of 111 *N*. *kaiseri* individuals based on 779 base pairs of the mitochondrial D-loop region. A total of 11 polymorphic sites were recorded including 10 transitions, one transversion and 10 variable characters were parsimony informative. Means nucleotide composition were A: 30.1%, T: 35.1%, C: 20.6% and G: 14.2%. The dominant haplotype (H5) of *N*. *kaiseri* was different up to 8 nucleotide substitutions with the rest of haplotypes. Haplotype diversity values (h) ranged from 0 in DMA, DAR and CH, to 0.71 in TZ population (Table 1). Nucleotide diversity values (π) varied from 0 to 0.001, and show a close relationship among the haplotypes (Table 1). The average number of nucleotide differences was 3.664 and the average number of nucleotide substitutions per site was 0.9±0.738. The mean of pairwise distance between all haplotypes was 0.83%. The results of the Independent Sample *t*-test (2-tailed) show that there wasn’t a significant difference in nucleotide (*t* = 0.72, df = 8, *P* = 0.49) and haplotype (*t* = 0.71, df = 8, *P* = 0.49) diversities between northern and southern regions. Pairwise uncorrected Kimura 2-parameter genetic distances between all populations (Table 3) in different regions ranged from 0–1.3% and for haplotypes were 0.1–1.5%. The genetic distance between two clades of *N*. *kaiseri* is 1.00±0.4%.

**Table 3:** K2P genetic distances values among 10 populations of *Neurergus kaiseri*.

No	Population name	1	2	3	4	5	6	7	8	9
1	**Abejdan**									
2	**Talezang;**	0.000								
3	**Dej Mohamad Alikhan**	0.000	0.000							
4	**Daregol**	0.013	0.013	0.013						
5	**Hajibarikab**	0.012	0.013	0.012	0.000					
6	**Kerser**	0.012	0.013	0.012	0.000	0.000				
7	**Choobeh**	0.013	0.013	0.013	0.000	0.000	0.000			
8	**Pifeh**	0.012	0.013	0.012	0.000	0.000	0.000	0.000		
9	**Bozorgab**	0.011	0.011	0.011	0.001	0.001	0.001	0.001	0.001	
10	**Tafo**	0.012	0.012	0.012	0.000	0.000	0.000	0.000	0.000	0.001

Phylogenetic relationships are represented by ML and Bayesian trees of seven haplotypes of *N*. *kaiseri* (Fig 2). The two methods of phylogenetic reconstruction gave similar tree topologies, although the statistical support of some nodes depended on which optimization criterion was used. The analyses produced highly concordant trees, each revealing that *N*. *kaiseri* forms a monophyletic lineage with respect to the congeneric species *(N*. *microspilotus* and *N*. *strauchii*) and *Triturus karelinii*. In the Bayesian tree (Fig 2), all haplotypes grouped into two main groups (the southern: TZ, DMA and AB, and the northern: KER, DAR, CH, TAF, BOZ and PIF).

**Fig 3:**
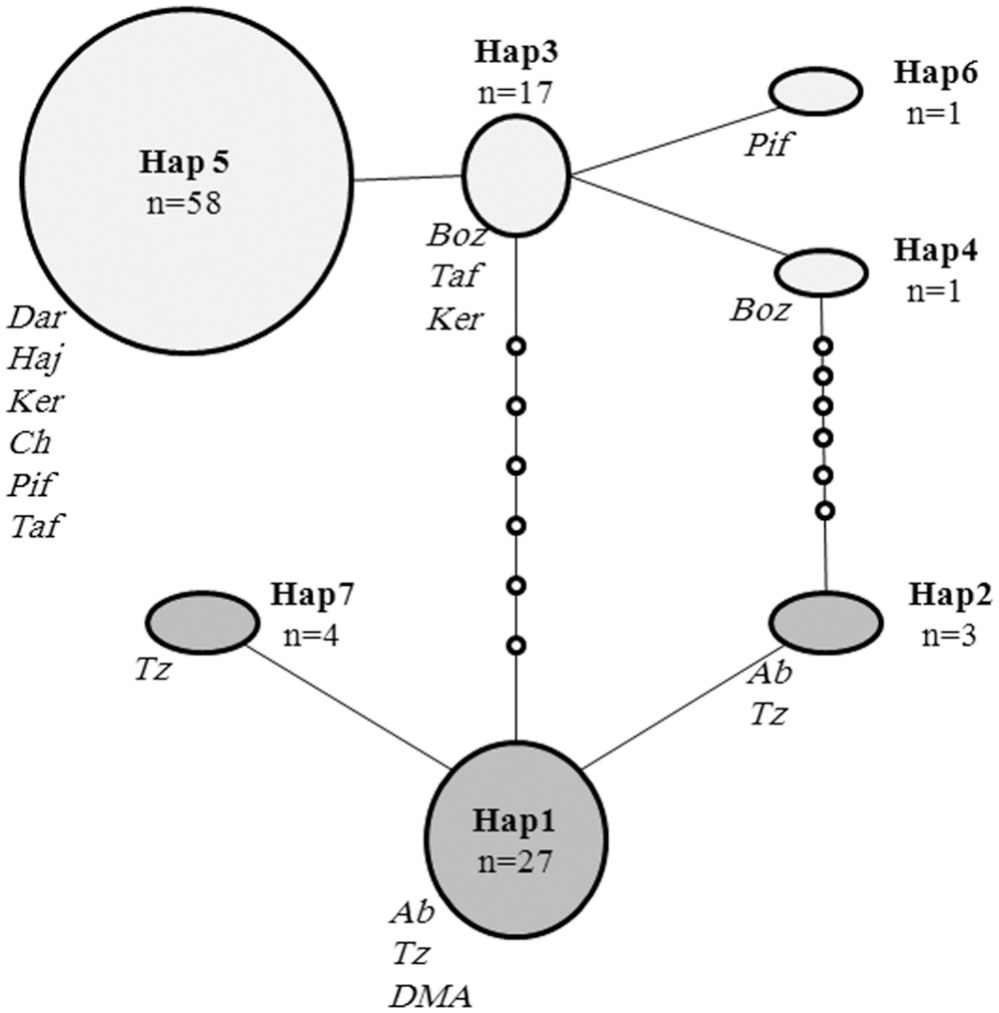
Statistical parsimony network of seven *N*. *kaiseri* haplotypes, obtained from 111 individuals. Red circles represent sampled haplotypes from the northern region and blue circles represent sampled haplotypes from the southern region. The letters codes of the populations are indicated in italic. The size of each circle is proportional to the relative frequency of that haplotype among all samples. Small circles indicate inferred haplotypes that are extinct or were not detected in analyses.

The statistical parsimony haplotype network (Fig 3), based on 95% statistical parsimony, suggested two haplotype subnetworks, which were in agreement with the topology described in the Bayesian tree. Of the 7 different haplotypes in all populations, 3 are found in 91.89% of the individuals. Of these, two haplotypes (5 and 3) are in the northern region and one (1) in the southern region. Given the greatest value for outgroup weight [44] haplotype 3 (found only in northern populations) was identified as the most likely ancestral haplotype. Haplotype 5 was the most widespread, shared among six of the ten locations (all in northern region, sites KER, DAR, CH, TAF, PIF and HAJ; Fig 1, Table 1), and occurred with the greatest frequency (75.32%) in the northern region. Haplotype 1 was the most abundant haplotype (79.41%) in the southern region and was found at all sites (AB, TZ and DMA). Interestingly, none of the haplotypes are shared between northern and southern regions. Shared haplotypes only occurred within regions (Fig 1) and we did not uncover any haplotypes shared between northern and southern regions. The haplotype network (Fig 3) showed a pattern suggestive of little or no gene flow between regions because northern and southern haplotypes were not intermingled.

The AMOVA results showed significantly genetic differences attributed to all hierarchical levels tested and in the three population groupings considered (within regions analyzed separately, among two regions “North and South” and among the populations without considering regions; Table 2). Results suggest that most genetic variation was significantly explained by differences among regions (94.31% genetic variation, θCT = 0.94, P < 0.01) and genetic difference was detected among populations within regions (2.69% genetic variation, θSC = 0.47254, P < 0.001; Table 2).

### Ecological comparisons between regions

**Fig 4:**
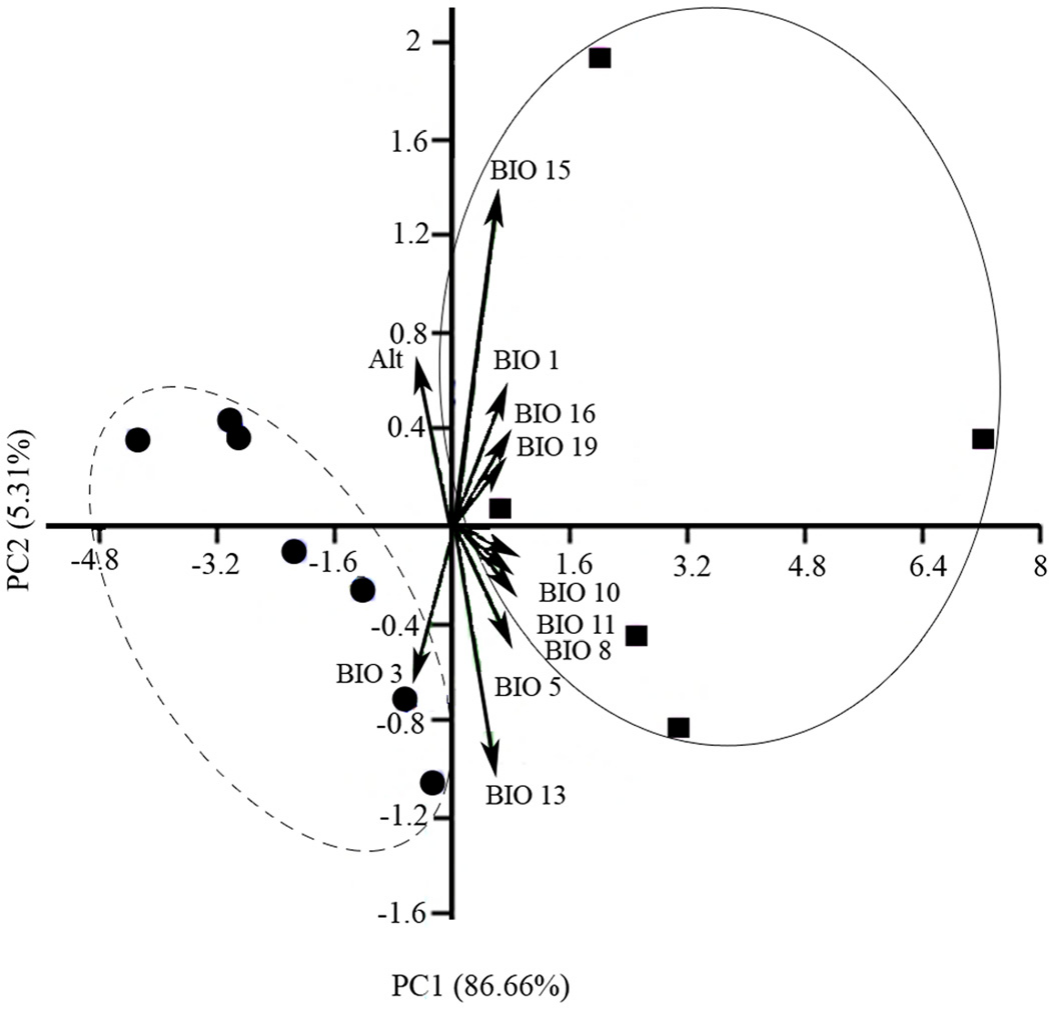
Plot of PCA based on 13 environmental variables describing 12 *Neurergus kaiseri* localities. The populations in the northern (dark circles) and southern (dark squares) regions were separated along the PC1 and PC2. The first two axes explain 86.66% and 5.31% of variation among regions. See Table 2 for environmental variable codes.

The first two principal components (PC1 and PC2) explained 86.66% and 5.31%, respectively, of the total variation and clearly separated the northern and southern localities along temperature and precipitation gradients and revealed a northern and a southern group (Table 4, Fig 4). The relative contributions of the different climatic variables to PC1 and PC2 are illustrated in the PCA distance biplot (Fig 4). Mean temperature of driest quarter, mean temperature of warmest quarter, mean temperature of wettest quarter, and mean temperature of coldest quarter with PC1 and precipitation seasonality with PC2 were highly positively correlated (Table 4). The 12 occurrence sites were divided into two clearly separated environmental spaces in the Cartesian coordinates formed by the first two principal components. The results show that northern populations occupy habitats that are cooler with lower winter temperatures and higher summer rainfall. In contrast, southern populations are characterised by warmer habitats with higher winter temperatures and wetter winters.

**Table 4:** Pearson correlation coefficients between 13 environmental variables and principal component axes.

Environmental variables		Principal component		
		PC1	PC2	PC3
BIO 1	Annual Mean Temperature	0.935***	0.199**	0.186**
BIO 3	Isothermality (P2/P7)*(100)	-0.835**	-0.230**	0.003**
BIO 5	Max Temperature of Warmest Month	0.966***	-0.159***	-0.170***
BIO 8	Mean Temperature of Wettest Quarter	0.973***	-0.167***	-0.085***
BIO 9	Mean Temperature of Driest Quarter	0.986***	-0.063***	-0.131***
BIO 10	Mean Temperature of Warmest Quarter	0.985***	-0.038***	-0.117***
BIO 11	Mean Temperature of Coldest Quarter	0.971***	-0.087***	-0.156***
BIO 13	Precipitation of Wettest Month	0.773**	-0.369**	0.511**
BIO 15	Precipitation of Seasonality (Coefficient of Variation)	0.843**	0.505*	0.034*
BIO 16	Precipitation of Wettest Quarter	0.964***	0.128***	0.119***
BIO 19	Precipitation of Coldest Quarter	0.963***	0.098***	0.099***
Alt	Elevation	-0.948***	0.247***	0.172***
Eigenvalue		10.40	0.637	0.441
% of variance		86.66	5.31	3.68

*** significance at the 0.1% nominal level,

** significance at the 1% nominal level,

* significance at the 5% nominal level

#### Association between genetic and environmental variation

**Fig 5:**
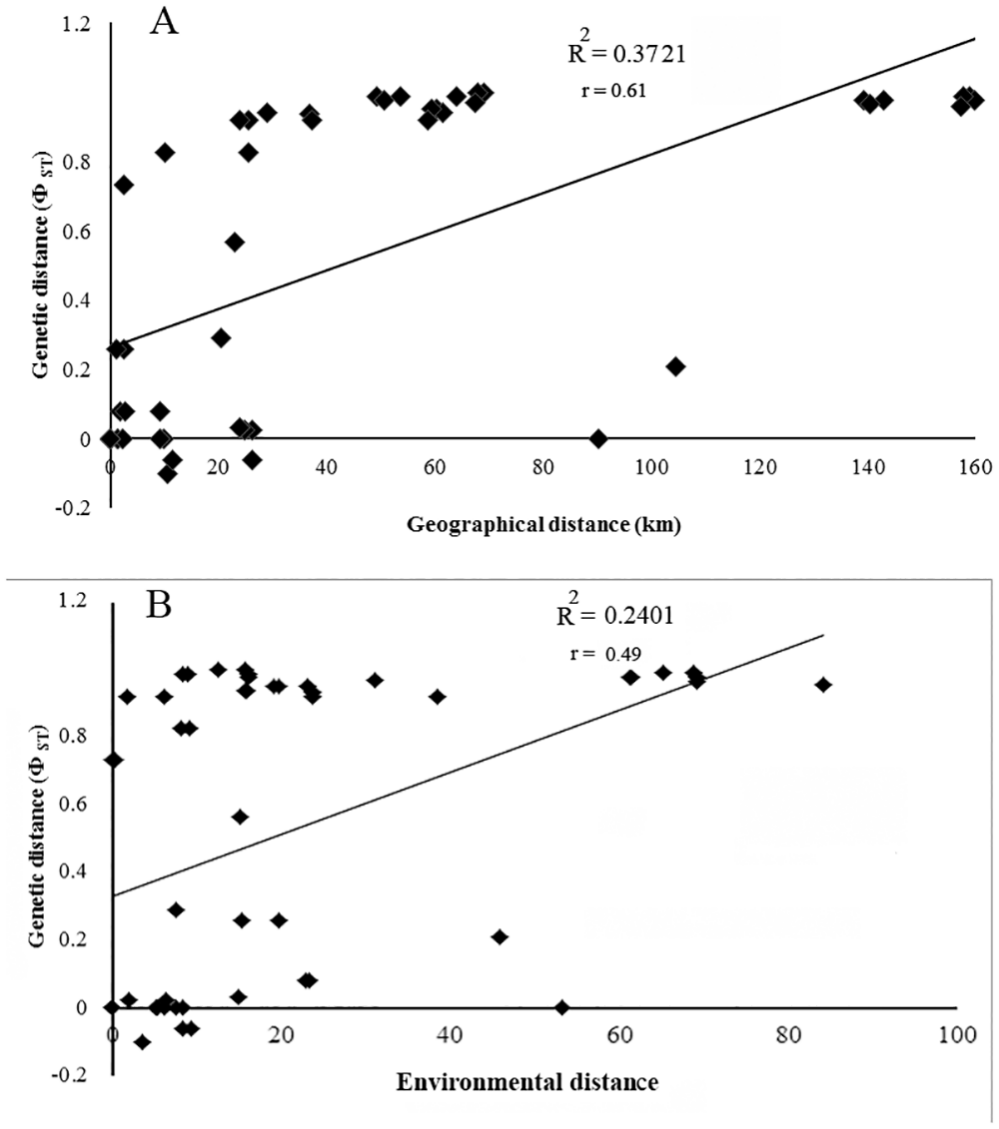
Plot of simple Mantel test showing the relationships between (A) geographic and genetic distances and (B) environmental and genetic distances among 10 populations of *Neurergus kaiseri*.

The analyzed populations revealed significant levels of isolation by distance (r = 0.61, P = 0.002; Fig 5A) and lower correlation between genetic divergence and environmental distance (r = 0.49, P = 0.023; Fig 5B). The correlation between genetic and geographical distances remained significant (r = 0.61, P = 0.002) even after accounting for the effect of environmental distance in a three-way Mantel test. On the other hand, the removal of the effect of geographical distance in the partial Mantel test resulted in a non-significant correlation between genetic and environmental distances (r = -0.364, P = 0.992).

## Discussion

As discussed by Steinfartz et al. [28], patterns of genetic variation can be effectively assessed by sequencing the complete D-loop region in urodeles. Other authors have also used the mtDNA D-loop for studying population genetic diversity [45] and conservation genetics [46] of different species of newts and have shown remarkable levels of genetic structuring that has been helpful to describe the relationships between newt populations and provides sufficient information to resolve historical divergences. In the present study, variation in the D-loop region has revealed notable levels of genetic structuring in *N*. *kaiseri*.

The distribution of the newt’s haplotypes demonstrated a high geographical structuring. The geographical structure found in the haplotype distribution which may result from a degree of isolation among populations within regions in north and south regions of the distribution range of *N*. *kaiseri* is also supported by hierarchical analysis of genetic diversity (Table 2). Steinfartz et al. [28], who have worked on phylogeny of salamandrid taxa including all species of the genus *Neurergus*, have presented a molecular clock calibrated for the D-loop indicating 0.8% sequence divergence per 1 Myr. Based on this time scale separation of the two species of the genus *Neurergus*, *N*. *microspilotus* in western and *N*. *kaiseri* in the southern Zagros Mountains occurred approximately 4 MYA. Accordingly, the two clades observed in *N*. *kaiseri* in the present study have been isolated about 1–1.25 million years.

The genetic analysis results in present study agree with the expectations of low genetic diversity within populations and high genetic differentiation between populations (θ_ST_ = 0.94) due to limited mobility over land in restricted ranges reported for other species of stream breeding amphibians [47]. Our combined analysis of genetic data and geographic distance (Fig 5A) suggests that in *N*. *kaiseri* with a very small distribution an important genetic variation exist which may have been shaped by isolation by distance rather than by ‘‘isolation by environmental distance”. There are reports of several species of salamanders with low mobility and strong fidelity to their breeding habitats with a genetic structure shaped under the influence of the Pleistocene glaciation [48,49]. Although the southern edge of the Zagros Mountains at 32 degrees latitude has been well away from direct influence of the glacial ice, indirect effects of glaciations by lowered temperature, increase in aridity, expansion of alpine glaciation and changes in hydroperiod of highland streams could have great impacts on distribution and abundance of amphibians [50]. Following glacial maxima a subsequent period of better climatic and hydrological conditions, leading to typical warm and humid interglacial periods, could have promoted a secondary range expansion with migration of individuals from south to the northern part of the species range [51]. Further genetic studies are needed to show that separation of the two clades in *N*. *kaiseri* could have been caused by isolation of populations in two refugia or changes in dispersal pattern of *N*. *kaiseri* under the influence of the adverse climatic conditions during the Pleistocene.

**Fig 6:**
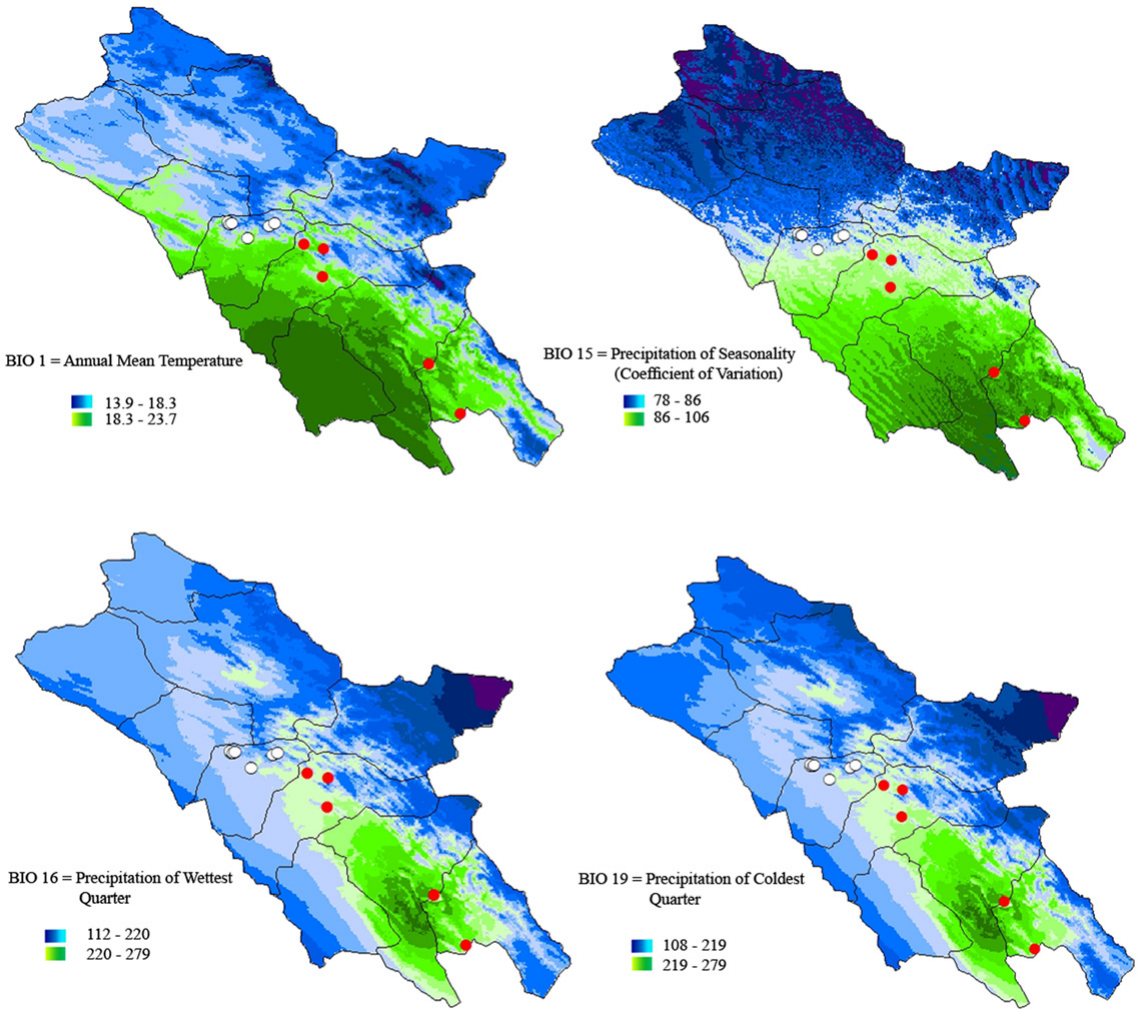
Different environmental variables in the distribution range of *Neurergus kaiseri*. A: Bio 1 (annual mean temperature), B: Bio 2 (precipitation of seasonality (coefficient of variation), C: Bio 16 (Precipitation of Wettest Quarter), D: Bio 19 (Precipitation of Coldest Quarter). Populations in the northern region represented by open circles and in the southern region represented by red circles.

As the climatic factors used in the principal components analysis (Fig 4) are highly correlated, separate climatic profiles of the distribution range of *N*. *kaiseri* including northern and southern populations were compared (Fig 6). Fig 6 demonstrates the variation in different bioclimatic variables in the distribution range of *N*. *kaiseri* such as annual mean temperature (A), precipitation of seasonality (B), precipitation of Wettest Quarter (C), and precipitation of Coldest Quarter (D). As is shown in Fig 1 northern and southern populations are located fairly isolated in different bioclimate maps.

The detected IBD pattern in this study explained the genetic divergence better than the IBED pattern (37% vs. 24%, respectively). Mantel tests across 10 populations detected low but significant correlations between genetic distance and geographic distance (r = 0.61, P = 0.002). However, similar correlation between genetic distance and environment distance was not significant (r = 0.49, P = 0.023). Such geographic isolation may partly be enforced by rough topography in a very steep and dry terrain causing limitation in gene flow among northern and southern population of *N*. *kaiseri* [26]. Limited gene flow in many amphibian species with limited dispersal abilities, strong site fidelity and spatially disjunct breeding habitat has been reported [43] including a small home range in their breeding streams for adult *Neurergus microspilotus* a closely related species to *N*. *kaiseri* occurring in western Zagross Range [52].

The Mantel tests showed positive, but statistically not significant, correlation between genetic and environmental distances for all populations. Contrary to our expectations of observing an effect of two disjunctive environments of more humid habitats at higher elevation (northern region), and drier habitats at lower elevations (southern region) on the spatial genetic structure of *N*. *kaiseri*, our study found no clear and significant correlation between genetic and environmental variable. The absence of a clear genetic differentiation might suggest that high gene flow among newts in breeding streams is still maintained despite habitat differentiation. There are recent evidences that support such gene flow in amphibians because of their ability to migrate over longer distances than previously presumed (e.g. [53,54,55]). It is also possible that *N*. *kaiseri* with high longevity of up to 14 years [56] living in a very small distribution range (789km^2^) with a small nearest neighbor distance 11.84 km [26] avoid or delay the effect of genetic drift.

According to the results obtained from present study an association exists between geographic distance and population genetic structure in the Endangered Kaiser’s Mountain Newt, *N*. *kaiseri*. Effect of habitat heterogeneity on the genetic structure of *N*. *kaiseri* cannot be ignored, but it is not strong enough to lead to a clear independent conclusion. Further studies based on more appropriate genetic markers and ecological studies are needed to better understand the underlying causes and mechanisms leading to the observed genetic structure. These studies should be based on larger sample size and use of more, already developed microsatellite loci [57] to test the effects of habitat heterogeneity on genetic structure. Such studies should also include the role of habitat alteration on population genetics of species with different density, dispersal ability and longevity.

## Conclusions

This study demonstrates the clear reciprocal monophyly obtained from the D-loop region with the lack of shared haplotypes between southern and northern populations. Genetic structure determined in the present study shows a clear historical phylogenetic pattern supported by recent genetic exchange as evidenced by the distribution of haplotypes among populations within and between the regions. Positive and significant correlation between geographic and genetic distances following controlling for environmental distance suggests a possible impact of geographic divergence shaping the genetic variation. Combination of genetic analysis and environmental data demonstrates a clear ‘‘isolation by distance” pattern and has provided broad conservation benefits as a combined approach that can provide valuable information regarding the likelihood studies for re-introduction programs or designating a protected area for an endangered species.
